# Depression in Rheumatoid Arthritis: Prevalence and Effects on Disease Activity

**DOI:** 10.3390/jcm13072058

**Published:** 2024-04-02

**Authors:** Cătălina-Elena Ionescu, Claudiu Costinel Popescu, Mihaela Agache, Georgiana Dinache, Cătălin Codreanu

**Affiliations:** 1 Rheumatology Department, “Carol Davila” University of Medicine and Pharmacy, 050474 Bucharest, Romania; catalina.ionescu@reumatologiedrstoia.ro (C.-E.I.); mihaela.agache@reumatologiedrstoia.ro (M.A.); georgiana.dinache@reumatologiedrstoia.ro (G.D.); catalin.codreanu@reumatologiedrstoia.ro (C.C.); 2 “Dr. Ion Stoia” Clinical Center of Rheumatic Diseases, 030167 Bucharest, Romania

**Keywords:** rheumatoid arthritis, depression, mental health, disease activity

## Abstract

**Background:**The primary objective of this study was to estimate depression’s prevalence in a cohort of rheumatoid arthritis (RA) patients, and the secondary objective was to evaluate the impact of depression on disease activity over time. **Methods:** We included all patients with RA presenting to our clinic from 2019 to 2020, who had three follow-up visits available. Depression prevalence was calculated using the patient’s history of diagnosed depression, and disease activity was assessed using the disease activity score for 28 joints (DAS28) and its components: tender joint count (TJC), swollen joint count (SJC), pain value on a visual analog scale (VAS), and inflammatory markers. **Results:** A total of 400 RA patients were included, 75 of whom had diagnosed depression, generating a prevalence of 18.8%. The mean values of DAS28 and its components were higher, with statistical significance, in the depression subgroup at all three follow-ups (*p* < 0.001). **Conclusions:** Depression is prevalent in the RA population, and leads to higher disease activity in dynamic evaluations. Assessing depression could be a psychological marker for RA prognosis with an important outcome in controlling disease activity.

## 1. Introduction

Rheumatoid arthritis (RA) is an inflammatory systemic chronic disease with diverse extra-articular manifestations and several strong associations with comorbidities, of which depression is among the most common [1]. The prevalence of depression in the general population is approximately 6%, while the prevalence among RA patients is significantly higher (16.8% [2]), even compared to other chronic diseases such as diabetes, Parkinson’s disease, or cancer [3]. Diagnosing depression in patients with RA poses some challenges because it has been proven quite challenging to distinguish depression from the normal emotional reaction to a chronic painful and debilitating disease. Additionally, RA patients may present constitutional symptoms frequently encountered in depression, such as fatigue, weight loss, insomnia, and lack of appetite. The overlap of depression in inflammatory immune-mediated diseases has been recognized for some time [4]. Different studies showed that immune-mediated inflammation affects and modulates neurogenesis, neurotransmission, neuroendocrine activity, and neuroplasticity [5].

Depression seems to influence RA disease activity and evolution: it has been shown that RA patients suffering from depression have worse disease prognosis over time, more severe pain, significant fatigue, higher functional deficit, more comorbidities, a higher rate of mortality, increased healthcare resource utilization, and globally lowered quality of life [6]. Psychological distress modulates overall health status by influencing different behaviors like treatment adherence, smoking, and physical activity. Depression lowers treatment adherence [7] and raises morbidity and mortality [8], partly by raising suicide risk [9]. It is also important to highlight the fact that depression in RA leads to higher addressability and medical costs [10], either directly by raising hospitalization numbers or indirectly by lowering work productivity [7]. Depression in RA leads to a globally lowered quality of life in these patients, a fact that was reflected in all of the domains of quality of life questionnaires [11]. Depression and anxiety have been incriminated as risk factors for RA flares [6] and they exert a major impact on the way patients perceive their current health status. Depression modifies a patient’s perception of their global health status and, as a consequence, lower thresholds of pain are quantified in higher disease activity scores, which further complicates RA management [12,13]. In patients with depression, pain can be persistent even if they receive disease-modifying antirheumatic drugs (DMARDs), so treating depression could potently raise pain thresholds for a better treatment response, which would ultimately lead to better quality of life. Multiple reports have observed a clear direct correlation between depression severity and RA disease activity. Higher depression scores are associated with higher disease activity in RA. As most of these studies were observational studies, they could not demonstrate causality or direction [14]; however, data regarding this affirmation are beginning to accumulate, especially following new discoveries about the common pathogenic features that these two distinct entities, RA and depression, share. Studies in early RA found that the higher the number of affected joints at disease onset, the higher the depression scores at 6 months [14]. In 2011, Kekow et al. revealed that patient-reported outcomes and depressive symptoms are improved by clinical remission among RA patients with active severe disease [15]. This relationship between patient-reported outcomes, depressive symptoms, and fatigue was initially studied in other literature reports [16,17,18]. In 2013, a systematic review conducted by Rathbun and colleagues found only seven studies that examined the longitudinal relationships between depression and RA outcomes. The main reason for the lack of data was not only the limited number of studies, but also their poor quality, so the review concluded that depression may worsen RA disease activity and treatment response [19]. It was previously reported that increased psychological distress predicts increased disease activity (measured via the 28-joint Disease Activity Score; DAS28) and reduced odds of reaching clinical remission over a 2-year follow-up period. However, this research is limited by its sub-standard identification of psychological distress, and its use of clinical trial data representing a relatively homogenous group of patients [20,21]. Patients with extreme baseline depression showed significantly increased levels of follow-up DAS28 in comparison with patients with no depression/anxiety at baseline. In comparison with patients who were never depressed/anxious, patients with depression/anxiety symptoms 50% of the time or at all time points reported significantly increased DAS28 scores at follow-up [21]. These results support previous systematic review findings that depression/anxiety influences long-term disease activity [19]. The most probable explanation is that depression and RA disease activity have a bidirectional relationship.

Depression is a strong negative predictor of remission after 3–6 months of DMARD therapy in clinical trials [22]. Randomized controlled trials (RCTs) with tocilizumab and conventional synthetic DMARDs (csDMARDs) have demonstrated that depressed RA patients had a 40% lower chance of achieving remission. Unfortunately, antidepressive treatment does not seem to produce a significant change in disease activity score. On the other hand, there are accumulating observations concerning psychological interventions, including cognitive behavioral therapy (CBT), that have positive effects on pain, fatigue and the global evaluation of RA disease activity [23].

It is thus apparent that depression in RA patients lowers remission rates by affecting subjective, but also objective, measurements of RA activity. Promoting early psychological well-being could prove cost-effective in the long-term management of RA, preventing higher levels of disease activity later and, thus, the need for more aggressive therapeutic strategies [24]. On the one hand, RA patients with depression seem to have lower functional scores, and on the other hand, depression treatment leads to functional status improvement [13]. A Danish study demonstrated a directly proportional relationship between depression symptoms and the functional status of RA patients [25]. Studies show that the Health Assessment Questionnaire (HAQ), which measures the global well-being of RA patients, is a strong predictor of psychological distress early in the evolution of RA. Also, demographic and socio-economic factors such as profession and financial status are associated with depression among RA patients. Fragoulis et al. reported that baseline depression score, unemployment, lack of partners, young age, HAQ, and higher RA disease activity are associated with the Hospital and Anxiety Disease Score (HADS) in depression. In their model, HAQ and baseline depression score most accurately predicted long-term depression scores. The association between HAQ score and depression is also supported by data from the Scottish Early RA study (SERA cohort) [14], which observed that depression independently predicted functional deficit after 12 months of the diagnosis of RA. The strong association of HAQ scores and depression was also observed in a multi-ethnic cohort [14].

In this context, the current study aims to highlight the importance of a correct and complete management of depression in RA patients by estimating the prevalence of depression in an RA cohort and by evaluating its effects over time on RA disease activity.

## 2. Materials and Methods

### 2.1. Patients and Inclusion

The study included all patients aged 18 years or above who were diagnosed with RA and who had at least three consecutive visits to our tertiary rheumatology clinic between 2019 and 2020. An RA diagnosis was made according to each attending senior rheumatologist and it was retrieved electronically based on International Classification of Diseases (ICD) codes. Depression was defined as either a specific ICD code in the electronical medical records of the patients (all coded by the different psychiatrists who diagnosed depression in these patients) or if the patients were taking specific antidepressants (namely, tricyclic antidepressants, selective serotonin re-uptake inhibitors, selective serotonin and norepinephrine re-uptake inhibitors, and/or monoamine oxidase inhibitors, all prescribed by their psychiatrists). All patients gave written informed consent at each visit regarding medical management and the use of medical data.

### 2.2. Medical Evaluations

All patients had at least three visits to the clinic in the 24-month study time frame, each 6 months apart from each other. Electronic records were used to retrieve demographic data (age, sex, smoking status), RA clinical variables (disease duration, TJC, SJC, patient global assessment on VAS, functional stage, pharmacological agents: glucocorticoids, csDMARDs, bDMARDs, tsDMARDs), local laboratory data measured using standard commercial kits (RF: 0–30 IU/mL, anti-CCP antibodies: ELISA 0–20 IU/mL, CRP: 0–5 mg/L, ESR: 0–30 mm/h), and radiographic staging information. The DAS28 was calculated with 4 variables (TJC, SJC, VAS, and CRP) and disease activity was defined as remission (DAS28 < 2.6), low (2.6 ≤ DAS28 ≤ 3.2), moderate (3.2 < DAS28 ≤ 5.1), and high (DAS28 > 5.1) [26,27]. On the first evaluation, patients were formally questioned regarding the dates of depression symptoms and diagnoses, as well as current and previous antidepressants. On the third evaluation, patients were questioned about whether their depression improved after RA treatment (yes/no) and whether their RA improved after depression treatment (yes/no).

### 2.3. Statistics

The normality of the data distribution was assessed using descriptive statistics, normality, stem plots and leaves, and Kolmogorov–Smirnov tests corrected by Lilliefors. Continuous variables are reported as “mean ± standard deviation” if normally distributed, or as “median (interquartile range)” if non-normally distributed, while dichotomous variables are reported as “observed frequency (percentage of group or subgroup)”. Mann–Whitney U tests were used to evaluate differences in the continuous variables between subgroups of dichotomous categorical variables, while associations between categorical variables were studied using χ^2^ tests or Fisher tests. The difference in variation of continuous variables (DAS28, TJC, SJC, VAS, ESR, CRP) between the three assessment times according to the presence of depression was assessed non-parametrically using Friedman tests with pooled comparisons. Statistical tests were considered significant if *p* < 0.035 for an alpha level of 0.05 and Bonferroni correction in order to avoid the type I error. Statistical analysis was performed using IBM SPSS Statistics version 25.0 for Windows (IBM Corp., Armonk, NY, USA).

## 3. Results

### 3.1. Demographic Data and RA Phenotype

The study included 400 RA patients (Table 1), of whom 75 patients (18.8%) were also diagnosed with depression, with only 16 patients (4% of the depression subgroup) undergoing pharmacologic antidepressive treatment. The sample and its subgroups included mostly women and the prevalence of active smoking was significantly higher in the depression subgroup. RA phenotype (disease duration, functional and radiographical stages, auto-antibodies) did not vary significantly between the depression and non-depression subgroups (Table 2).

### 3.2. Baseline Evaluation

At baseline (visit 1), there were no notable differences in the DAS28 among the RA patients with depression and without depression (Table 3). Notably, approximately 79.9% of RA patients without depression and 78.6% of RA patients with depression were outside the DAS28-defined target of activity (moderate and high disease activity).

Regarding the time relationship between RA and depression, of the 75 patients with depression, only 37 (49.3%) provided complete answers, 2 refused to answer the depression questions, and 36 patients offered only scarce data (statistically unsuitable). Of the 37 patients who answered, 9 (24.3%) developed depression after RA and 28 (75.7%) developed RA after depression.

### 3.3. Follow-Up Evaluations

**Figure 1:**
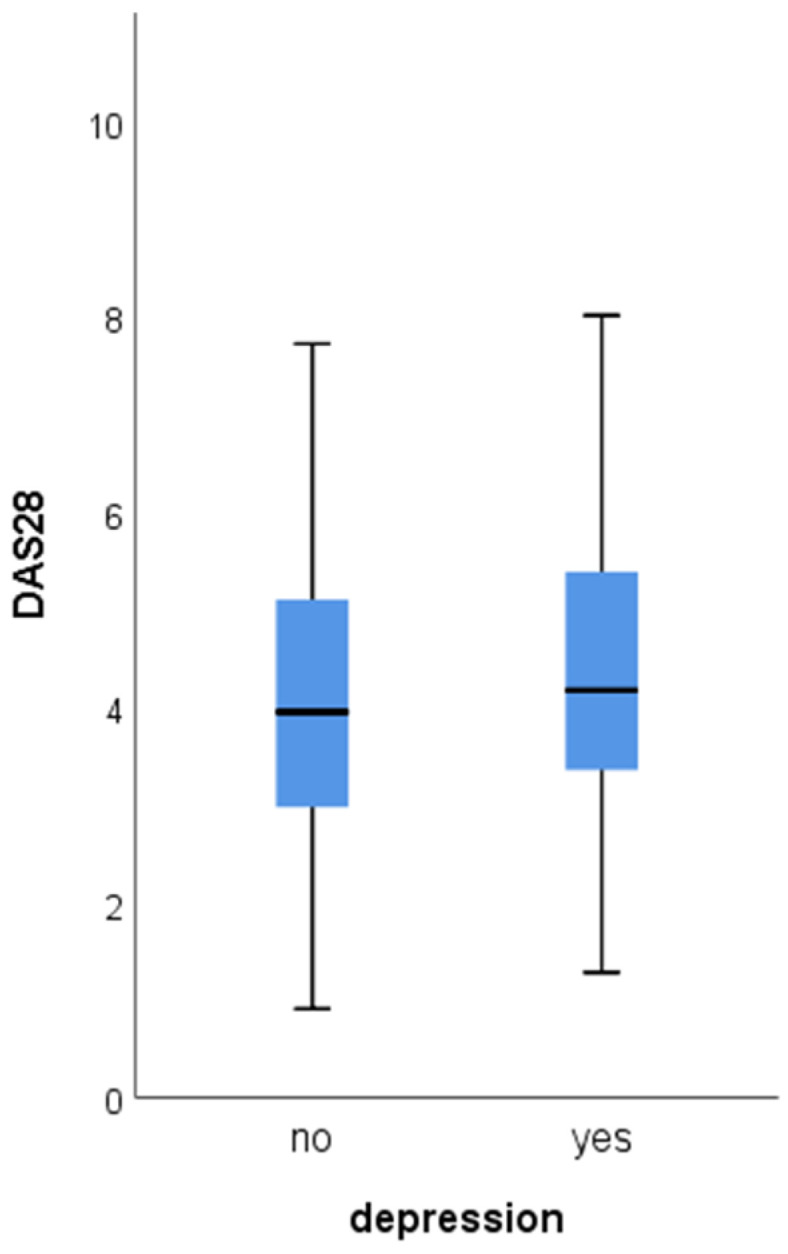
Difference of mean DAS28 at the second follow-up depending on the presence of depression (4.5 ± 4.2 vs. 4.1 ± 3.9, *p* = 0.045).

**Figure 2:**
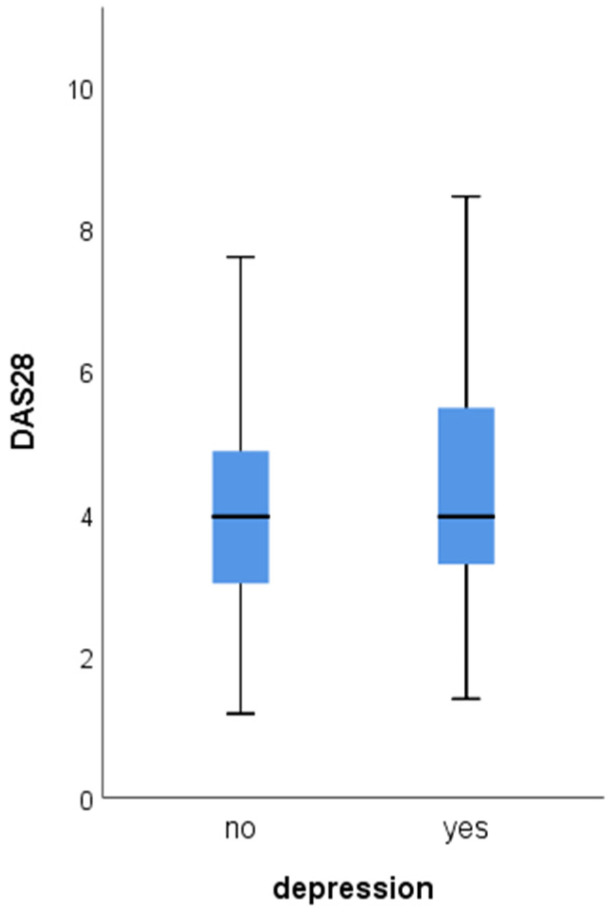
Difference of mean DAS28 at the third follow-up depending on the presence of depression (4.4 ± 1.6 vs. 4.0 ± 1.3, *p* = 0.038).

At the second and third follow-ups, RA patients with depression had higher mean DAS28 (Figure 1 and Figure 2). At each visit, all of the DAS28 components (TJC, SJC, ESR, and CRP) were significantly higher among RA patients with depression (Table 4). The frequency of antirheumatic drugs (csDMARDs, bDMARDs, tsDMARDs, glucocorticoids) did not differ significantly among the subgroups on both follow-up evaluations.

Of the 37 complete answers, 15 (40.5%) patients declared that treating their depression improved RA symptoms and 18 (48.6%) patients declared that treating their RA improved depression symptoms.

## 4. Discussion

The observed prevalence of depression in our RA cohort was 18.8%. A similar percentage was reported by a 2013 meta-analysis, in which depression affected 16.8% of RA patients [23]. The majority of the patients in our depression subgroup were women, and the literature identifies female sex as a potential risk factor for depression [28]. Approximately 8% of our RA patients with depression were active smokers, while in the non-depression subgroup, only 2.8% were smokers (*p* = 0.032). Depression is a known risk factor for smoking [2]. In the literature, a longer disease duration is associated with depression [29]. Our data also indicated a longer RA disease duration in depressed patients, but the difference was not statistically significant.

At all three time points, the RA disease activity according to DAS28 was significantly higher in depressed RA patients. Data concerning the way that depression leads to higher disease activity in RA, quantified by DAS28, are scarce and contradictory. There are studies that have demonstrated an independent and proportional correlation between depression and RA activity: higher depression scores are associated with higher RA activity scores. Most being observational studies, causality or its direction cannot be demonstrated [14]. The mechanism by which depression has been associated with higher DAS28 was reported to be the increased subjective components of the score (TJC, VAS) [13]. In the present study, TJC showed a statistically significant higher value at all three time points in depressed patients compared to non-depressed patients, a fact that was expected. The SJC, considered a more objective component of the clinical examination, showed statistically significant higher values in all three evaluations of our study, which would suggest the presence of clinical inflammation in dynamic evaluations of patients with depression. There are also studies that suggest that there is a close relationship between depression and inflammation, considering the fact that CRP levels have been correlated with depression scores at multiple dynamic assessments. Also, a higher CRP level at baseline makes an apparent distinction between patients with RA who will ultimately develop depression [14]. In our study, both CRP and ESR show higher values in all three evaluations in patients with depression compared to those without depression, with statistical significance for ESR.

Our results and the literature review point out the importance of further studies in this interdisciplinary field, especially because depression is prevalent among RA patients and studies have clearly demonstrated that affected patients have a poorer long-term prognosis. The correlation between RA and depression is strong, regardless of the deterministic direction, so mental health should be addressed routinely, including in randomized clinical trials. Depression assessment in this context could be a psychomarker for the rheumatological prognosis. Depression should be assessed before and after starting DMARDs, given that there is plausible evidence that depression treatment could also improve physical illnesses. Since the variation in DAS28 determines therapeutic decisions and the variation in depression scores influences DAS28, it follows that depression influences therapeutic decisions. Therefore, diagnosing and managing depression is necessary for optimal RA management. Unfortunately, interventional studies testing this hypothesis are lacking. Such studies should evaluate the effect of depression treatment on the prognosis of RA. Furthermore, interventional studies are needed to identify the optimal pharmacologic and psychiatric therapy of depression in RA patients.

In interpreting the results of our study, the reader should bear in mind two important limitations, namely, the retrospective design and the fact that it captures real-world data with non-standardized diagnoses of RA and depression, which were based on expert opinion. Also, there were insufficient data to assess the causal and temporal relationship between RA and depression, a subject that is currently under development in a prospective study design.

## 5. Conclusions

Depression is prevalent among RA patients and, compared to RA patients without depression, it is associated with a higher prevalence of smoking and higher disease activity indices (overall DAS28 and its components, including objective measures of SJC and acute-phase reactants) over several evaluations and despite similar treatment. Further studies are needed both to evaluate a causal relationship between RA and depression and to assess the impact of depression treatment and prevention on RA outcomes.

## Figures and Tables

**Table 1:** Demographic characteristics of RA patients.

		Depression		
	All (n = 400)	No (n = 325)	Yes (n = 75)	*p*
age (years)	64.8 ± 11.5	65.0 ± 11.7	63.8 ± 10.5	0.401
women	86.3%	84.6%	92.0%	0.107
active smokers	3.8%	2.8%	8.0%	0.032

**Table 2:** RA phenotype.

		Depression		
	All (n = 400)	No (n = 325)	Yes (n = 75)	*p*
RA duration (y)	11.0 (12.0)	10.0 (12.0)	12.0 (14.0)	0.364
functional stage 1	13.0%	12.9%	13.3%	0.622
functional stage 2	60.0%	60.6%	57.3%	0.622
functional stage 3	25.8%	24.6%	29.3%	0.622
functional stage 4	1.2%	1.9%	0	-
radiographic stage 1	18.0%	18.8%	18.7%	0.967
radiographic stage 2	52.0%	52.3%	58.7%	0.944
radiographic stage 3	20.5%	20.6%	10.7%	0.578
radiographic stage 4	9.5%	8.3%	12.0%	0.578
+ RF	71.8%	72.3%	69.3%	0.579
+ anti-CCP	66.8%	67.7%	62.7%	0.366
+ RF and anti-CCP	59.0%	60.0%	54.7%	0.381

CCP—cyclic citrullinated peptide; RF—rheumatoid factors; RA—rheumatoid arthritis; y—years; + positive.

**Table 3:** Baseline evaluation of RA patients.

		Depression		
	All (n = 400)	No (n = 325)	Yes (n = 75)	*p*
DAS28	4.6 ± 1.6	4.6 ± 1.6	4.8 ± 1.7	0.282
remission	9.8%	10.4%	12.9%	0.557
LDA	8.5%	9.7%	8.6%	0.768
MDA	36.0%	43.1%	28.6%	0.027
HDA	35.5%	36.8%	50.0%	0.042
≥1 csDMARD	92.5%	92.9%	90.7%	0.508
b/tsDMARD	31.3%	33.3%	22.7%	0.073
glucocorticoids	35.8%	35.9%	37.3%	0.821

b/csDMARD—biologic/conventional synthetic disease-modifying antirheumatic drug; DAS—disease activity score; LDA, MDA, HDA—low, moderate, or high disease activity; ts—targeted synthetic; RA—rheumatoid arthritis.

**Table 4:** The variation of DAS28 and its components.

	Visit 1		Visit 2		Visit 3		
	Depression		Depression		Depression		
	No (n = 325)	Yes (n = 75)	No (n = 325)	Yes (n = 75)	No (n = 325)	Yes (n = 75)	*p*
DAS28	4.6 ± 1.6	4.8 ± 1.7	4.1 ± 3.9	4.5 ± 4.2	4.0 ± 1.3	4.4 ± 1.6	<0.001
SJC	2 (6)	3 (8)	1 (3)	2 (6)	1 (4)	1 (4)	<0.001
TJC	6 (8)	7 (9)	4 (7)	7 (7)	4 (8)	5 (7)	0.020
VASp	38 (46)	50 (40)	27 (32)	40 (60)	29 (40)	50 (40)	<0.001
ESR	38 ± 24	42 ± 28	34 ± 23	38 ± 28	31 ± 25	33 ± 27	<0.001
CRP	18 ± 29	24 ± 40	15 ± 24	19 ± 33	14 ± 25	41 ± 23	0.070

Notes: DAS28, ESR, and CRP are expressed as “mean ± standard deviation”, while the others as “median (interquartile range)”; significant differences depending on the time point (1, 2, or 3): DAS28 1–3 and 1–2; SJC 1–3 and 1–2; TJC 1–3; VASp 1–3 and 1–2; ESR 1–3 and 2–3, respectively; *p*-values represent the significance of the Friedman non-parametric tests; abbreviations: CRP—C-reactive protein (mg/L); DAS—disease activity score; TJC—tender joint count; SJC—swollen joint count; VASp—visual analog scale patient (mm); ESR—erythrocyte sedimentation rate (mm/h).

## Data Availability

The data presented in this study are available on request from the corresponding author due to patient confidentiality.
